# Air, Skin, and Biological Monitoring of French Fire Instructors’ Exposure to Particles/PAHs During Controlled Fire and Mitigation Strategies

**DOI:** 10.3390/toxics13020106

**Published:** 2025-01-28

**Authors:** Pauline Zangl, Clément Collart, Renaud Persoons

**Affiliations:** 1 Service Départemental d’Incendie et de Secours (SDIS) Isère, 38000 Grenoble, France; pauline.zangl@sdis38.fr (P.Z.); clement.collart@sdis38.fr (C.C.); 2 Grenoble Teaching Hospital, EPSP-TIMC Laboratory, Université Grenoble Alpes, 38400 Grenoble, France

**Keywords:** firefighting instructors, controlled fire, PAHs, particulate matter (PM), biomonitoring, skin absorption

## Abstract

Occupational exposure as a firefighter was recently classified as carcinogenic to humans by the IARC. Fire instructors’ exposure to carcinogenic PAHs is a major concern, and studies that have tried to assess the determinants of their exposure are scarce. An air and biomonitoring study was conducted in fire instructors performing simulated training exercises in enclosed containers. Air samples were collected, as well as urine samples from 22 firefighting instructors, and skin wipes were collected from FFs’ skin at the end of the exercises. PAH metabolites (1-hydroxypyrene, 3-hydroxybenzo(a)pyrene, 2/3-hydroxyfluorene, and 2/3-hydroxyphenanthrene) were measured in urine samples at three sampling times (beginning of shift, end of shift, and next morning). Airborne PAHs were dominated by low molecular weight compounds (naphthalene), and levels were as high as 67 µg·m^−3^ close to the containers, decreasing at higher distances. Skin contamination was observed both on the neck/face and hands/wrists of fire instructors and pilots. Ten times lower skin contamination was observed when nitrile undergloves were worn. High internal exposure was measured, with 1-hydroxypyrene and 3-hydroxybenzo(a)pyrene levels frequently exceeding maximum recommended values in occupational settings (up to 2.8 µmol/mol creatinine for 1-OHP, 14 µmol/mol creatinine for ΣOH-PAH, and 1.0 nmol/mol creatinine for 3-OHBaP), whereas benzene exposure was revealed to be very low. These types of exposure were found to derive both from dermal absorption (combustion products deposited on the skin) and inhalation (when removing SCBA outside the containers). Several recommendations are proposed in order to reduce both exposure routes (nitrile undergloves and half-masks in the vicinity of containers), harmonise decontamination (PPEs) and cleaning procedures, and prevent the dermal absorption of PAH from turnout gear. This study emphasises the complex PAH exposure profiles of fire instructors and characterises the main drivers of exposure, highlighting the need for better mitigation strategies.

## 1. Introduction

Occupational exposure as a firefighter (FF) was recently classified as carcinogenic to humans based on sufficient evidence for mesothelioma and bladder cancer in humans [1]. Limited evidence was also found for other cancers such as colon, prostate and testicular cancers, melanoma, and non-Hodgkin lymphoma. In addition, recent findings have shown a convincing body of evidence that occupational exposure to wildfires or prescribed burns have both acute and possibly longer-term respiratory effects among FFs, including reduced lung function, increased airway dysfunction and inflammation, upper/lower respiratory tract symptoms or increased asthma incidence [2]. Occupational exposure as a firefighter is highly complex and involves a broad range of hazards including wood smoke, combustion products (polycyclic aromatic hydrocarbons (PAHs), fine particles and volatile organic compounds (VOCs)), building materials, perfluorinated/polyfluorinated substances (PFASs) contained in firefighting foams or used on turnout gear, flame retardants, and diesel exhaust [3]. In a recent review, 31 carcinogens were identified and quantified in 49 papers relating to occupational exposure to carcinogens among wildland FFs [4]. Only 3 of these 49 studies concerned French FFs [5,6,7], highlighting the lack of data on the occupational exposure of French FFs. A recent study focused on Portuguese wildland FF airborne exposure to fine particles, PAHs and metalloids and measured levels below available occupational limits [8]. Other studies have highlighted the potential for occupational exposure to PAHs in some fire station areas with PAH exposure being related to fire suppression and exposure to contaminated gear and trucks [9].

FF exposure is highly variable, depending on FFs’ employment and tasks (full- versus part-time FFs or volunteer FFs), as well as on the type of fires (wildland fires, urban fires or structure fires). Among these job categories, less attention has been paid to fire instructors, who play a critical role in the training of FFs. Fire instructors are often involved in multiple simulated fire training exercises on a given day with various types of fuel being used (plywood, chipboard, oriented strand board, pallet, straw, etc.). Live fire or simulated fire training scenarios involve either unconfined open-flame fires or controlled fires in confined containers. Cumulative exposure to air contaminants exceeding that of FFs in operational fire environments can be experienced by fire instructors [10]. The dermal uptake of combustion products from the microenvironment inside an instructor’s turnout gear has rarely been studied, although skin absorption may significantly contribute to the overall exposure of instructors [1]. Little data have been published on the occupational exposure of fire instructors to combustion fumes and PAHs. A (rare) French study evaluated the level of benzene and PAH contamination in fire instructors, demonstrating low urinary levels of PAH metabolites but high ambient airborne levels with possible skin contamination [7]. PAH levels have rarely been measured in ambient air or personal air during simulated controlled compartment fires or training exercises (live fires) in international studies. Total PAH levels range from 2200 to 180,000 µg·m^−3^ in ambient air and from 1.8 to 4.8 µg·m^−3^ in personal air. Among the 16 PAHs classified as priority pollutants by the U.S. Environmental Protection Administration (EPA), the levels of the seven priority pollutants (benzo(a)anthracene, chrysene, benzo(a)pyrene, benzo(b)fluoranthene, benzo(k)fluoranthene, dibenz(a,h)anthracene, and indeno(1,2,3-cd)pyrene) designated by the EPA as Group B2 (classified as probable human carcinogens) were low with concentrations in the range of 150–17,490 µg·m^−3^ in ambient air but only 0.02–0.24 µg·m^−3^ in personal air [1].

Despite growing evidence of the percutaneous absorption of fireground contaminants in both in vitro [11] and real-life studies [12], only a few research projects have so far focused on the use of biomarkers to assess occupational exposure and its associated effects on firefighting instructors. Rossbach et al. observed that live fire training was associated with additional PAH uptake, and that dermal absorption was assumed to be the major exposure route due to the use of SCBA [13]. Fent et al. observed a 30-fold increase in 1-hydroxypyrene levels for instructors following a single day of oriented strand board exercises and assumed that dermal absorption probably contributed to the biological levels since the respiratory route was well protected [14]. More recently, Lang et al. used 1,2-dihydroxynaphthalene (LMW naphthalene metabolite) to prove dermal naphthalene absorption during training [15]. So far, no biomonitoring study has attempted to characterise the internal exposure of FF instructors to carcinogenic BaP along with air samples and skin wipes.

The objectives of this study were therefore to characterise external and internal PAH exposure of (the less studied) French fire instructors during simulated fire training in confined containers using untreated wood panels, to assess skin contamination, and to identify the main drivers of their exposure in order to ensure that operational protocols adequately protect fire instructors.

## 2. Materials and Methods

### 2.1. Population Characteristics

The study population (Table 1) consisted of 22 fire instructors categorised into four different jobs: fire instructors (n = 13) and pilots (n = 4) entering the maritime containers, and supervisors (n = 4) and logisticians (n = 1) remaining outside. The fire instructors wore full turnout gear with self-contained breathing apparatus (SCBA) during training exercises inside the containers. The pilots were in charge of igniting fires and refuelling fire sites with pallets of untreated wood. The supervisors remained outside the containers and managed safety procedures. The logistician was responsible for supplying the suits and PPE to the FFs (firefighting gear, gloves, helmets, SCBA) and handling used PPE at the end of the training exercises. Most of the workforce was composed of men, and only three subjects reported that they were smokers (4–6 cigarettes per day). A variety of underglove types were used by the FFs (latex, vinyl, nitrile), although 32% did not wear any undergloves. The fire instructors and pilots always wore SCBA while they were inside the containers, whereas only some of them used a half-mask (FFP3 filter, no gas cartridge) while they remained outside in the vicinity of the containers. Only 26% of the supervisors (who remained outside the containers) used a half-mask (FFP3) in the vicinity of the containers. All the FFs took a shower at the end of the day, and some of them also took a shower before lunch. Underpants and tee-shirts (long sleeved) were changed twice per day (at the start of each training session).

At the time of the study, the fire instructors were allowed to perform simulated fire-training exercises for a maximum of three consecutive days, which was limited to thirty days per year. Each working day included two training sessions, one in the morning and one in the afternoon, with an average duration of three hours each. The average time spent inside the containers by fire instructors each day ranged from 1 h 30 min to 3 h. The training sessions included reconnaissance and progression in a smoky environment, victim recovery and fire extinction phases. During the study, all training sessions were video recorded, and the videos were subsequently analysed using a measurement synchronisation platform for human behaviour analysis (Captiv^®^ software, Teaergo^®^, version 2.4.56) in order to precisely describe working habits, the wearing and removal of PPE and decontamination procedures. The following variables were studied: wearing of undergloves (with or without), wearing of respiratory protective equipment (RPE) (SCBA or half mask (FFP2) or none), location and activity (debriefing, rest, decontamination) as well as the tasks carried out during cleaning and decontamination (washing of face, hands or protective personal equipment (PPE)).

### 2.2. Air Monitoring Protocols

Ambient air monitoring was carried out during several exercises (medium to high generation of hot smoke) in order to measure concentrations of fine particles (PM_1_, PM_2.5_ and PM_10_) and PAHs (both particulate high molecular weight PAHs and gaseous low molecular weight PAHs). PM was sampled using an AirBeam2^®^ particle counter detector (HabitatMap^®^, Paris, France) employing a light scattering method, whereas PAHs were sampled using portable sampling pumps and dedicated sorbents. The PAH sampling protocol has been described elsewhere [16]. Briefly, gaseous and particulate PAHs were analysed using an Alliance 2695 HPLC system coupled to a 2475 Multi λ fluorescence detector (Waters⁠^®^, Paris, France). Separation by chromatography was performed by injecting 30 μL into a PAH C18 column (Waters⁠^®^ PAH C18 3 × 250 mm, particle size: 5 μm) with an acetonitrile/water gradient and a column oven temperature set to 32 °C). Seventeen PAHs were sampled, including 15 of the 16 PAHs on the priority list established by the US-EPA (naphthalene, acenaphthene, fluorene, phenanthrene, fluoranthene, pyrene, benzo(a)anthracene, chrysene, benzo(e)pyrene, benzo(a)pyrene, benzo(b)fluoranthene, benzo(j)fluoranthene benzo(k)fluoranthene, dibenzo(a,h)anthracene, benzo(g,h,i)perylene) + indenopyrene. Acenaphthylene was excluded due to very poor fluorescence sensitivity, and two other PAHs (benzo [e]pyrene and benzo [j]fluoranthene) were added. Fluoranthene and pyrene were sampled in gaseous and particulate phases due to their presence in gas and dust. PAHs were collected using a combined aerosol–vapour sampler connected to a pump regulating the flow rate at 1 L·min^−1^. A 37 mm diameter Teflon filter (pore size: 0.2 μm) was used to trap particulate PAHs and was connected in series to an Amberlite XAD2 cartridge used to trap gaseous PAHs. PAHs adsorbed into the XAD2 cartridge were extracted by elution using 2 mL of acetonitrile. Ten millilitres of dichloromethane were added to each Teflon filter and then placed in an ultrasonic extractor for 120 min followed by a rotary evaporator at 30 °C. HPLC coupled to fluorescence detection was used for analysis with external calibration. The LOQs ranged from 0.05 to 0.2 ng·filter^−1^ for particulate PAHs and 0.05–0.3 ng·tube^−1^ for gaseous PAHs, approximately corresponding to 0.2–1 ng·m^−3^ for a 4 h sample.

The sampling duration ranged from 1 to 4 h for PM_1/2.5/10_, and it was 4 h for PAHs. Sampling pumps and real-time monitors were positioned outside the containers at increasing distances: 0 m (immediate vicinity), 1 m, 3–5 m (rest area), 6 m (stretcher area), 10 m (debriefing area) and 30 m (decontamination area).

### 2.3. Skin Wipe Sampling

Surface skin samples (wipes) were collected from all FFs at the end of the training sessions before the performance of cleaning/decontamination protocols. Skin wiping of the neck/face and hands/wrists was performed to quantify PAH deposition on the skin. Skin wiping was performed in accordance with the French INRS MetroPol HAP M-448 method [17]. Briefly, the skin (10 × 10 cm) was wiped twice using the same wipe containing 1 mL of acetone. Skin wiping was performed by the same trained occupational health physician for all subjects. Wiping was performed on the face and then on both sides of the neck (one wipe), followed by another wipe performed on the hands (both sides) and wrists (one wipe), resulting in two wipes per subject. Blank field wipe samples consisted of one unused wipe. The wipes were placed in 15 mL screw-cap glass vials and stored frozen (−20°C) in the dark until extraction. (Particulate) PAHs were extracted from wipes using a 50/50 dichloromethane/methanol mixture (15 mL), ultrasonicated and further analysed using the same method as for air monitoring. Quality controls (PAH-spiked blank wipes) were regularly used in order to ensure the accuracy of the results.

### 2.4. Biomonitoring Protocols

Urine samples were collected between April and June 2023 at three sampling times: at the start and end of the working day following one to three consecutive days of simulated fire-training exercises and 16 h later (next morning). Before the start of the study, all workers gave their informed consent to participate in the study, and urine samples were collected as part of regular biomonitoring campaigns organised by the occupational health physicians. Samples were rapidly refrigerated and stored/shipped frozen to the medical laboratory. The following biomarkers were determined: 1-hydroxypyrene (1-OHP, pyrene metabolite), OH-PAH (2-hydroxyfluorene, 3-hydroxyfluorene, 2-hydroxyphenanthrene, 3-hydroxyphenanthrene), 3-hydroxybenzo(a)pyrene (3-OHBaP, BaP metabolite) and S-phenyl mercapturic acid (S-PMA), benzene metabolite). ΣOH-PAH was referred to as (2-Fluo + 3-Fluo + 2-Phen + 3-Phen). PAH analyses were performed following the enzymatic deconjugation of PAH metabolites and liquid–liquid extraction (LLE) or solid-phase extraction (SPE). 1-OHP and 3-OHBaP were determined by LC fluorescence using a method described elsewhere [18]. OH-PAH was determined by GC-MS-MS using a method described elsewhere [19]. S-PMA analyses were performed following acidic deconjugation and determined by LC-MS-MS. The accuracy of the results was ensured by successful participation in external quality assessment schemes (G-EQUAS Germany: https://www.g-equas.de, accessed 18 December 2024). The quality controls (QCs) used were either commercially available QC (1-OHP) or home-made controls (blank urine spiked with commercial standards for 3-OHBaP). The limits of quantitation were 0.05 µg·L^−1^, 0.05 ng·L^−1^ and 0.1–0.2 µg·L^−1^ for 1-OHP, 3-OHBaP and OH-PAH, respectively. S-PMA, 1-OHP and OH-PAH analyses were carried out under ISO 15189 laboratory accreditation.

### 2.5. Statistics

Statistical analyses were performed using SPSS Statistics^®^ 26.0 software from IBM (Inc., Chicago, IL, USA). The normality of distributions was assessed using Shapiro and Kolmogorov–Smirnov tests, and both airborne PAHs and urinary PAH metabolites had skewed distributions. Log-transformed distributions were used to obtain Gaussian distributions of the study variables. The geometric mean (GM), geometric standard deviations (GSD), minimum (Min), maximum (Max) and quartiles were used for descriptive statistics. Analysis of variance (ANOVA) and the Kruskal–Wallis test were used for mean comparisons of PAH or 1-OHP/3- OHBaP/ΣOH-PAHs/S-PMA levels between groups. The Spearman ρ coefficient was used for correlation tests between quantitative variables. Urinary creatinine concentrations outside [0.3–3 g·L^−1^] were excluded from the dataset.

## 3. Results

### 3.1. PM and Airborne PAH Levels

Highly variable PM levels were measured, ranging from 1 to 335 µg·m^−3^ (PM_1_), 2 to 486 µg·m^−3^ (PM_2.5_) and 15 to 930 µg·m^−3^ (PM_10_), respectively. The most polluted areas were located in the immediate vicinity of the containers with median concentrations of 59 µg·m^−3^ (PM_2.5_) and 97 µg·m^−3^ (PM_10_), respectively. PM concentrations at greater distances (stretcher, debriefing and decontamination areas) were shown to be much lower with a 5-fold reduction for PM_2.5_ (Med: 12 µg·m^−3^) and a 3-fold reduction for PM_10_ (Med: 32 µg·m^−3^) at 10 m from the containers. The WHO 24 h reference thresholds of 15 µg·m^−3^ (PM_2.5_) and 45 µg·m^−3^ (PM_10_), respectively, were often exceeded both in the immediate vicinity of the containers and also at greater distances (5–10 m) on occasions. The proportion of PM_2.5_ within PM_10_ ranged from 14 to 52%. This proportion was higher (49%) near the containers than further away (34%). Similarly, the proportion of PM_1_ within PM_2.5_ was 67% on average. The maximum particulate concentrations were measured when the container doors were opened, reaching up to 300 µg·m^−3^ for PM_1_ and 600 µg·m^−3^ for PM_2.5_. At the location where used PPE was stored following exercises, PM_2.5_ levels reached 16 µg·m^−3^ and PM_10_ levels reached 217 µg·m^−3^.

**Figure 1:**
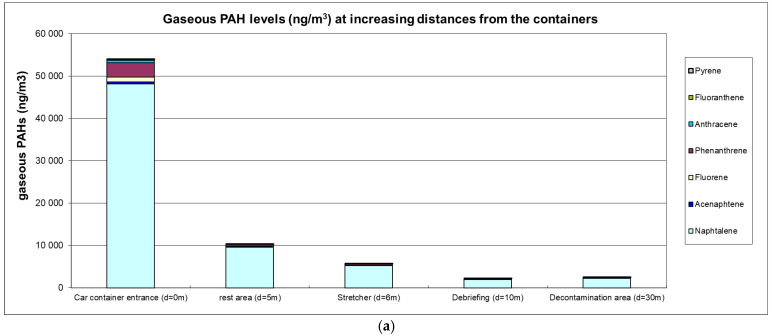

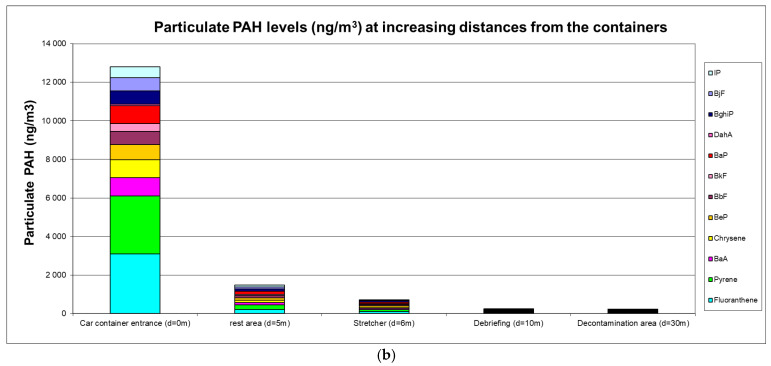
(**a**) Gaseous PAH levels (ng·m^−3^); (**b**) particulate PAH levels (ng·m^−3^).

The airborne PAH levels are presented in Figure 1a,b.

High ambient air levels were measured in the immediate vicinity of containers with levels reaching 53 µg·m^−3^ for gaseous PAHs (predominantly naphthalene) and 12.5 µg·m^−3^ for particulate PAHs (predominantly fluoranthene and pyrene). Carcinogenic BaP levels were 0.93 µg·m^−3^, and the sum of (proven or suspected) carcinogenic PAHs reached 4.75 µg·m^−3^. A clear decreasing trend at greater distances was observed with a 5-fold or 8-fold reduction at 5 m for gaseous or particulate PAHs, respectively. At a distance of 10 m from the containers, gaseous and particulate PAH levels were 25-fold and 54-fold lower, respectively.

### 3.2. Skin Wipes

**Figure 2:**
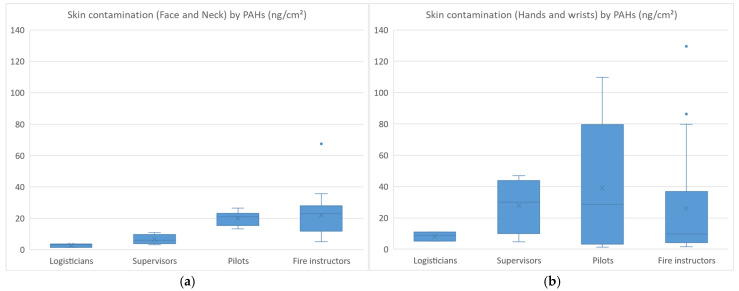
Skin contamination (face and neck (**a**)) (hands and wrists (**b**)) of firefighters by PAHs.

The skin contamination levels of FFs by PAHs are presented in Figure 2a,b.

The skin contamination of FFs (n = 21) ranged from 1 to 67 ng·cm^−2^ for face/neck (median values: 3; 6; 21; 23 ng·cm^−2^ for logisticians; supervisors; pilots; fire instructors) and from 1 to 129 ng·cm^−2^ for hands/wrists (median values: 9; 30; 29; 10 ng·cm^−2^ for logisticians; supervisors; pilots; fire instructors). Higher face/neck contamination was observed in pilots and fire instructors, which was consistent with their regular presence inside the containers. Hand/wrist contamination was also found to be higher in pilots and fire instructors, but similar contamination levels were also observed in supervisors (not wearing gloves) who sometimes helped their colleagues remove their used equipment. Fluoranthene and pyrene were the most abundant PAHs measured on skin wipes, both for face/neck and hand/wrist samples, which was consistent with the air monitoring results. On examination of skin contamination based on the type of undergloves worn, the median levels were 4.5 ng·cm^−2^ (nitrile), 9.6 ng·cm^−2^ (latex), 12.4 ng·cm^−2^ (no undergloves) and 44 ng·cm^−2^ (vinyl), respectively. The degree of skin contamination increased as follows: nitrile gloves (reference) < latex gloves or no gloves (×2–3) < vinyl gloves (×10). The skin of FFs was therefore shown to be 10-fold more contaminated when vinyl undergloves were worn than when nitrile gloves were worn.

### 3.3. Biomonitoring Results

**Figure 3:**
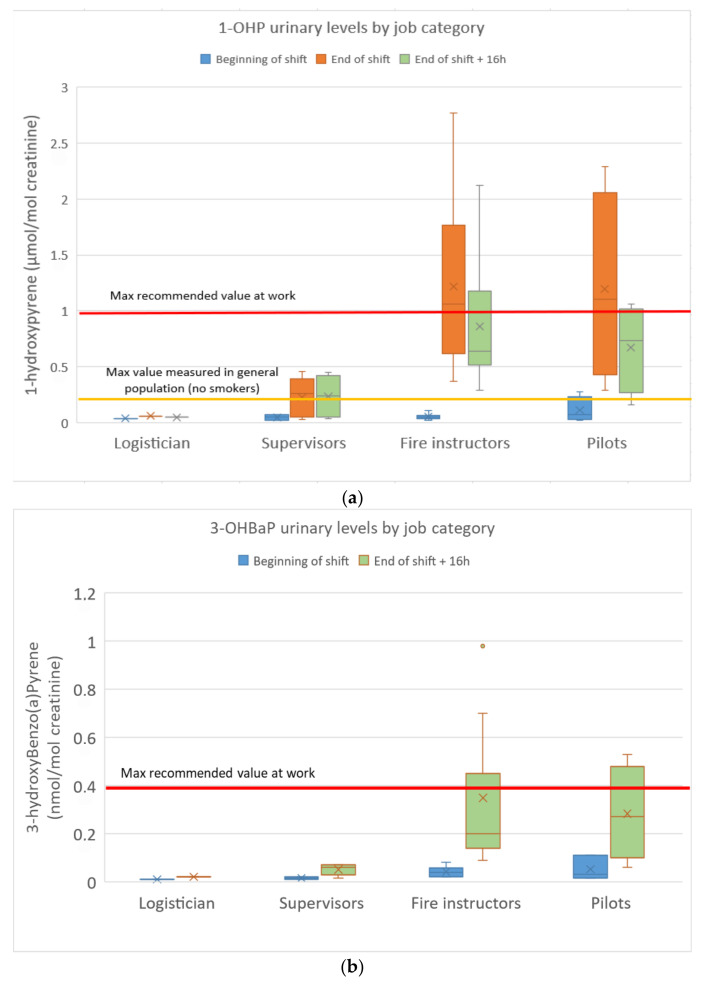

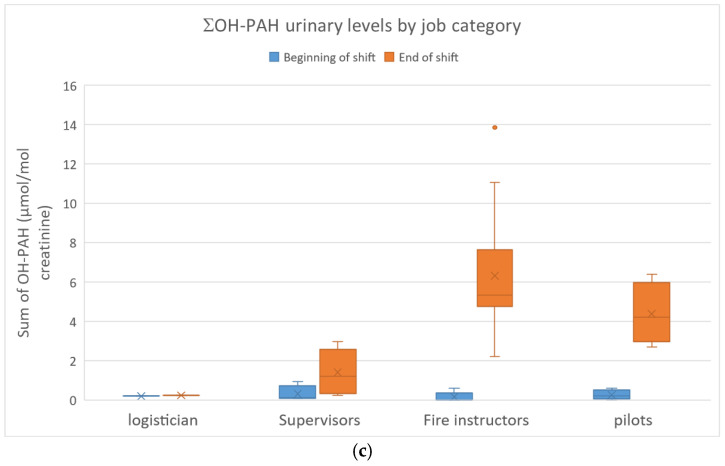
(**a**): 1-Hydroxypyrene urinary concentrations in FFs; (**b**): urinary 3-hydroxybenzo(a)pyrene concentrations in FFs; (**c**): sum of fluorene/phenanthrene (ΣOH-PAH) metabolite concentrations in FFs.

The urinary concentrations of pyrene, benzo(a)pyrene and fluorene/phenanthrene metabolites in the FFs are presented in Figure 3a–c.

1-OHP concentrations ranged from 0.02 to 2.77 µmol/mol of creatinine (n = 5/66 < limit of detection). Among the 22 FFs, nine values (seven fire instructors and two pilots) exceeded the maximum recommended value in occupational settings (1 µmol/mol of creatinine, highest concentration at which no genotoxic effects were found in the white blood cells of PAH-exposed workers). The fire instructors (GM: 1.0 µmol/mol) and pilots (GM: 0.9 µmol/mol) were found to be much more exposed to pyrene than the supervisors (GM: 0.12 µmol/mol) and the logistician. Most of the urinary 1-OHP levels exceeded the French reference value in the non-smoking population (0.52 µg/g of creatinine) as recommended by the French Public Health Agency *Sante Publique* (Esteban study 2014–2016). Baseline levels at the beginning of a shift (BS) were similar to those of non-occupationally exposed controls, not evidencing any chronic accumulation. Urinary 1-OHP levels 16 h after the end of a shift were only slightly lower than those measured at the end of the previous shift, indicating clear skin absorption (slower absorption through the skin with a delayed urinary elimination peak). No significant differences were observed between smokers and non-smokers. A 21-fold increase between end-of-shift and baseline (BS) 1-OHP levels was observed for fire instructors compared to a 13-fold increase for pilots and a 3-fold increase for supervisors.

3-OHBaP concentrations ranged from <0.01 to 0.98 nmol/mol of creatinine (n = 14/44 < limit of detection, all at BS). Among the 22 FFs, four values (three fire instructors and one pilot) exceeded the French maximum recommended value in occupational settings (0.4 nmol/mol of creatinine). The fire instructors (GM: 0.26 nmol/mol) and pilots (GM: 0.21 nmol/mol) were found to be more exposed to BaP than the supervisors (GM: 0.04 nmol/mol) and the logistician. Once again, baseline levels at the beginning of a shift (BS) were similar to those of non-occupationally exposed controls without any chronic accumulation. No significant differences were observed between smokers and non-smokers. A 7-fold increase between end-of-shift + 16 h and baseline (BS) 3-OHBaP levels was observed for fire instructors compared to a 6-fold increase for pilots and a 3-fold increase for supervisors. 

OH-PAH concentrations ranged from <0.1 to 7.03 µmol/mol of creatinine (n = 59/176 < limit of detection, 56/59 at BS). ΣOH-PAH (2-F + 3-F + 2-P + 3-P) ranged from <0.1 to 13.9 µmol/mol of creatinine. At ES, higher levels were observed in the fire instructors (Med: 5.7 µmol/mol of creatinine) and pilots (Med: 4.2 µmol/mol of creatinine). Base levels at the beginning of a shift (BS) were frequently below the limit of detection and therefore similar to controls. No significant differences were observed between smokers and non-smokers. A 57-fold increase between end-of-shift and baseline (BS) ΣOH-PAH levels was observed for fire instructors compared to a 19-fold increase for pilots and a 5-fold increase for supervisors.

Mean urinary S-PMA concentrations were, respectively, 0.17 µg·g^−1^ of creatinine at beginning of shift, 0.38 µg·g^−1^ of creatinine at end of shift (ES) and 0.25 µg·g^−1^ of creatinine at ES+16h (next morning). The maximum levels were 1.8 µg·g^−1^ of creatinine. The RAC ECHA biological limit value (BLV) of 2 µg·g^−1^ of creatinine was never exceeded, and the biological guidance value (BGV) of 0.5 µg·g^−1^ was only rarely exceeded by (non-smoker) fire instructors. No significant differences were observed between the exposure groups, but significantly higher levels were measured in smokers (GM: 1.32 µg·g^−1^) than in non-smokers (GM: 0.20 µg·g^−1^) (*p* < 0.01).

## 4. Discussion

This study aimed to characterise the airborne and skin exposure of firefighting instructors to particulate matter and PAHs during live fire training in container structures (smoke diving exercises). The results revealed high airborne PM_1_, PM_2.5_ and PM_10_ concentrations in the area around the containers (0–30 m) as well as high PAH levels. Concentrations measured at a distance of <5 m from the containers largely exceeded the maximum recommended values or occupational exposure limits for the pollutants in question (<15 µg·m^−3^ and <45 µg·m^−3^ recommended in WHO guidelines for PM_2.5_ and PM_10_ 24 h average exposures, respectively, <0.15 µg·m^−3^, as recommended by the French Health Insurance system for BaP). However, these concentrations did not reflect real exposures as the FFs wore SBCA while inside the containers with only short periods of exposure when removing their SBCA at the end of the training sessions. Consequently, the measured PM levels probably overestimated the 8 h averaged exposures encountered by fire instructors. However, they highlighted the need to wear RPE during overhaul/knockdown. PM_1_ was found to predominate in the mixture, raising the issue of FF exposure to nanoparticles (<0.1 µm) during live fire training exercises, and the SOPs to be implemented in order to prevent this. Emissions of fine particles that are mostly smaller than PM_2.5_ and generally in the nanometre to the submicron range have been reported for wildfires [1]. In addition, smoke, soot and particulate emissions vary greatly depending on fuel composition and fire conditions [20].

The PAH mixture in the air samples was dominated by low molecular weight (LMW) compounds, including naphthalene, fluorene, phenanthrene and pyrene. This profile is consistent with other studies showing LMW PAHs to be predominant in wood smoke emissions [13]. However, high molecular weight (HMW) PAHs were also identified at elevated levels, including carcinogenic BaP and EPA group B2 compounds, with concentrations, respectively, exceeding 0.9 µg·m^−3^ (BaP) and 4.5 µg·m^−3^ (Sum 7-B2 EPA) in the immediate vicinity of the containers. This PAH profile only represents fires burning untreated wood panels and may be different in other firefighting circumstances (treated wood, building materials, wildland fire). The total PAH concentrations measured in this study (2200 to 65,000 ng·m^−3^) are of the same order of magnitude as those measured in the ambient air during wildfires and prescribed burns (from 56 to 9103 ng·m^−3^) [1]. The maximum BaP levels measured in the immediate vicinity of containers (930 ng·m^−3^) exceeded the maximum peak values of 140 ng·m^−3^ during live wildfires observed in other studies [21].

Skin contamination measured in skin wipe experiments revealed both face/neck and hand/wrist contamination with similar skin PAH loads. Our results are consistent with those obtained in the study conducted by Fernando et al. in Canadian FFs during training exercises at burn houses, where the median skin loads for PAHs ranged from 50 to 120 ng·cm^−2^ and were similar at five different skin sites (fingers, back, forehead, neck, wrist) [22]. In an experimental study, Mayer et al. found that PAH contamination on filters under hoods in the neck region was higher than in samples taken under jackets in the chest region, highlighting the need to protect the neck site [23]. Interestingly, we observed much higher hand/wrist PAH contamination when vinyl undergloves were worn and much lower contamination when nitrile gloves were worn by fire instructors. Other authors have also recommended the use of nitrile butadiene rubber undergloves to reduce exposure to toxic contaminants in FFs despite the slightly increased risk of deeper burns [24]. There is also growing evidence that firefighters assigned to attack and search have higher post-fire median hand contamination than other positions [25]. In a previous study, the same authors already showed that firefighters wearing full protective outfits absorbed PAHs into their bodies through their skin with the neck being the primary site of exposure and absorption due to the lower level of skin protection afforded by hoods [26]. Our results confirmed that the neck is a source of skin contamination with PAHs and needs to be properly protected.

Our biomonitoring results revealed high internal exposure to PAHs but very low exposure to benzene. A clear effect of live fire training on urinary PAH (pyrene, BaP, fluorene, phenanthrene) metabolite excretion suggested that training exercises were a source of internal PAH exposure for fire instructors. Our results are higher than those published by Rossbach et al. in German firefighting instructors where median 1-OHP levels reached 0.4 µg·g^−1^ of creatinine (or 0.21 µmol·mol^−1^ of creatinine) at the end of training + 3 h [13]. They also exceed the urinary 1-OHP levels measured by Fent et al. in instructors during exercises involving combustion of pallets and straw (median: 0.8 µg·g^−1^ of creatinine and Max: 1.6 µg·g^−1^ of creatinine post-3rd exercise) [14]. Although other sources of exposure (tobacco smoke for n = 3 subjects, diesel exhaust) may have contributed to the overall exposure in our study, these sources were considered to be of little importance in comparison to occupational exposure (no significant differences between smokers and non-smokers, no barbecue the day of urine sampling). High urinary pyrene and BaP metabolite concentrations were frequently measured in this study, with one third of (End of Shift) 1-OHP levels exceeding recommended values in occupational settings, and most of them largely exceeding reference values in the general population. The ACGIH Biological Exposure Index (BEI^®^) guidance value of 2.5 µg·L^−1^ was frequently exceeded, but this value is assumed to be valid for chronically exposed workers with a typical pyrene/BaP ratio of 2.5. This value should probably be adjusted to take into account the current Pyrene/BaP ratio (between 3 and 4 in this study), resulting in an adjusted value of 3–4 µg·L^−1^ that would still be exceeded by a few fire instructors. 3-OHBaP urinary values also occasionally exceeded the maximum recommended value in a few fire instructors and pilots. In contrast, in the only available French biomonitoring study on fire instructors (n = 7) where 3-OHBaP was measured, most of the concentrations were below the limit of detection (<0.1 ng·L^−1^), but the FFs were extinguishing a fire from outside a small container [7]. The urinary ΣOH-PAH levels measured in this study were shown to be much higher than in non-smokers in the US NHANES survey [27], particularly for fire instructors and pilots. Our results were comparable to and of the same order of magnitude as those published by Rossbach et al. in firefighting instructors [13], but they were higher than those reported by Fent et al. [14]. Urinary naphthalene metabolites were not analysed in this study as they are highly influenced by smoking status and other types of environmental exposure, frequently resulting in concentrations found within non-occupationally exposed males [19].

The internal PAH exposure assessed in our study reflected only a short period of time (three consecutive days of work). Our results should be interpreted in light of the number of days of simulated fire-training exercises performed per year (≤30 days/year at the time of the study), since fire instructors are not exposed to PAHs on a daily basis. Consequently, the annual averaged internal exposure to PAHs might be much lower when taking into consideration periods without exposure. Biomonitoring studies combining different biomarkers of exposure and of effect are currently limited but would be very useful in order to improve risk assessment [28]. Analysis of urinary TetraolBaP, reflecting the carcinogenic pathway of BaP, would also be useful in addition to 3-OHBaP in FF biomonitoring studies, as this biomarker has been shown to be relevant in recent studies for workers occupationally exposed to PAHs [29]. In addition, future studies should also include in vitro/in vivo assays in order to conduct a more realistic health risk assessment and explore the relative contribution of skin exposure to the overall PAH internal dose [30].

PAH exposure was assumed to be mixed in this study, resulting from both the inhalation of combustion products following the removal of SCBA at the end of exercises (often in the vicinity of the containers in smoke plume) and skin absorption (both during fire training through fire suits and afterwards during the decontamination of turnout gear and cleaning). Inhalation exposure was assumed to occur only at the end of fire training. Our results highlight the need to protect fire instructors’ airways from particulates/PAHs during post-fire activities, as already suggested by Horn et al. [31].

The combination of two urine samples, collected at the end of a shift and the next morning, made it possible to identify skin as a major source of exposure. In fact, urinary 1-OHP excretion peaks tend to occur around 9 h following exposure in the event of major skin absorption compared to around 3 h after the end of a shift in the event of inhalation exposure [32]. Consequently, a biomonitoring strategy based on repeated samplings over a 24 h period can help differentiate between inhalation and/or skin exposure. Rossbach recommended 3-hour post-exposure sampling for fluorene/phenanthrene/pyrene metabolites and 1 h post-exposure sampling for naphthalene metabolites [13]. Other authors also recommend sample collection at a time point of 2 to 4 h after fire suppression in order to show a consistent, statistically significant pattern compared with baseline samples [33]. Our findings thus confirm the relevance of collecting several urine samples (end of shift and 2–6 h later) in order to reflect the toxicokinetics of PAHs and to capture the urinary elimination peaks of these various metabolites (faster elimination half-lives of LMW PAHs but longer ones for HMW PAHs).

Following this study, several recommendations were made in order to ensure better protection of FF instructors. These recommendations included the following:

- Shower as soon as possible in order to remove any residual skin contamination.
- Wash hands before eating to help reduce hand-to-mouth ingestion of chemical or biological contaminants.
- Use untreated pallets and straw only, but no OSB (Oriented Strand Board), which has been associated with higher emissions [14]; ideally use simulated smoke generation wherever possible.
- Use particle filter masks after removing SCBA in order to avoid inhaling particles/combustion products from contaminated protective gear after training.
- Wear disposable (nitrile) gloves during removal of protective equipment, and use nitrile undergloves only.
- Field decontamination using dish soap, water, and scrubbing in order to reduce PAH contamination on turnout jackets (−85% in Fent et al. study [25]) and cleansing wipes recommended to reduce PAH contamination on neck skin (−54%).
- Encourage laundering and wet soap preliminary exposure reduction methods (post-fire) that are effective to reduce surface contamination and appear to prevent accumulation of contamination after repeated exposure [34].
- Remove SCBA (at the end of exercises) at a greater distance (>10 m) from the containers and also hold debriefings at a distance of >10 m.
- Harmonise cleaning and decontamination procedures for all instructors, and promote strict skin hygiene to remove combustion products.

## 5. Conclusions

This study characterised both external (PM) and internal (PAHs, Benzene) exposure of French firefighting instructors working occasionally in enclosed containers during live fire training exercises. High airborne particle levels and high urinary PAH metabolite concentrations were observed, frequently exceeding maximum recommended values, along with clear skin contamination. The neck and hands were shown to be major areas of contamination, both during training exercises and afterwards, during cleaning/decontamination procedures. Wearing nitrile undergloves was associated with lower skin contamination in comparison to other gloves. Several recommendations were made in order to better protect FFs from exposure and prevent health risks, including more frequent showering, wearing of RPE during overhaul/knockdown, the use of nitrile undergloves, better cleaning/decontamination procedures, and regular safety information sessions.

## Figures and Tables

**Table 1:** Population characteristics.

Main Characteristics	Study Population n (%)
Gender	
Female	2 (9%)
Male	20 (91%)
Smoking status	
Smokers	3 (13.6%)
Non-smokers	19 (86.4%)
Age	
<35 years	5 (23%)
35–40 years	9 (41%)
>40 years	8 (36%)
Seniority as FF	
≤5 years	12 (55%)
>5 years	10 (45%)
Job categories	
Fire Instructors (FI)	13 (59%)
Pilots (P)	4 (18%)
Supervisors (SU)	4 (18%)
Logisticians (L)	1 (05%)
Undergloves	
Latex	4 (18%)
Vinyl	5 (18%)
Nitrile	6 (32%)
No undergloves	6 (32%)
Respiratory Protective Equipment (RPE)	
Fire Instructors (FI)	100% SCBA inside containers
Pilots (P)	100% SCBA or FFP3* half-mask inside containers
Supervisors (SU)	26% FFP3* half-mask/74% none
Logisticians (L)	None

* FFP3: Filtering FacePiece 3.

## Data Availability

The raw data supporting the conclusions of this article will be made available by the authors on request.
